# Hidden Y Chromosome Mosaicism in 48 Egyptian Patients with Turner's Syndrome

**DOI:** 10.1155/2013/463529

**Published:** 2013-07-28

**Authors:** Mervat M. El-Eshmawy, Sohier Yahia, Faeza A. El-Dahtory, Sahar Hamed, El Hadidy M. El Hadidy, Mohamed Ragab

**Affiliations:** ^1^Internal Medicine Department, Mansoura Specialized Medical Hospital, Faculty of Medicine, Mansoura University, Mansoura 35516, Egypt; ^2^Pediatric Department, Mansoura University Children Hospital, Faculty of Medicine, Mansoura University, Mansoura 35516, Egypt; ^3^Genetics Unit, Mansoura University Children Hospital, Faculty of Medicine, Mansoura University, Mansoura 35516, Egypt

## Abstract

*Background*. The presence of Y chromosome material in Turner's syndrome (TS) patients is a risk factor for the development of gonadoblastoma. Although conventional cytogenetic analysis is the definitive diagnosis of TS, low level Y chromosome mosaicism may be missed. Molecular analysis has demonstrated a higher proportion of mosaicism, but there is controversy regarding the prevalence of Y chromosome-derived material in those patients. *Aim and Methods*. This study was conducted to investigate the prevalence of hidden Y chromosome mosaicism in 48 TS Egyptian patients using polymerase chain reaction (PCR) for molecular DNA analysis of SRY gene and compare our results with those in the literature. *Results*. None of TS patients had a cytogenetically obvious Y chromosome; Y chromosome material was detected only at molecular analysis. SRY gene was found in 9 TS patients (18.75%) with the classical 45,X karyotype, whereas all other patients were SRY negative. *Conclusion*. Cytogenetically undetected Y chromosome mosaicism is common in TS patients; these data reinforce the need for adequate diagnosis of Y chromosome material in those patients. Molecular screening for Y chromosome-derived DNA should be routinely carried out in all TS patients.

## 1. Introduction

Turner's syndrome (TS) is one of the most common chromosomal abnormalities affecting 1 in 2500 newborn females [1]. It is characterized by short stature, gonadal dysgenesis, primary hypogonadism, congenital heart disease, renal anomalies, and a variety of somatic features [2]. TS was suggested to be due to absence of the second X chromosome in part or full [3]. 

The cytogenetic abnormality associated with TS was first described by Ford and coauthors in 1959 [4]. Since then, a variety of other karyotypic findings have been determined; classical 45,X is identified in about half of the patients, and the remaining half have either structurally abnormal sex chromosome, for example, 46,X,i(Xq) or are mosaic with other cell lines with normal (46,XX) or abnormal sex chromosomes [5]. In addition, a cell line containing the Y chromosome is present in 5% of patients, and further 3% of cases have an unidentifiable marker sex chromosome, presumably derived from a Y chromosome [6].

Y chromosome-specific SRY gene is one of the key genes involved in human sex determination. SRY gene encodes a testis specific transcription factor that plays a key role in sexual differentiation and development in males and is located on the distal region of the short arm of Y chromosome [7]. SRY expression initiates a network of gene activity that transforms the undifferentiated gonad, genital ridge into testis. 

Previous studies have confirmed that TS patients with a Y chromosome-derived material in their genome may develop gonadoblastoma later in life [8, 9], which may be as high as 30% [10]. In addition to gonadoblastoma, more invasive tumors such as dysgerminoma may also occur in Y chromosome carrying patients with gonadal dysgenesis [11].

Routine conventional analysis may miss Y chromosome. So, the use of molecular techniques in detecting presence of Y chromosome material is becoming increasingly important in determining those at risk of developing gonadoblastoma. Chu [12] stated that polymerase chain reaction (PCR) is more effective than conventional cytogenetic analysis for detecting hidden Y chromosome mosaicism.

*Aim of the Work*. The present study was conducted to investigate the prevalence of hidden Y chromosome mosaicism in TS patients and compare our results with those in the literature.

## 2. Subjects and Methods

Forty-eight TS patients aged 6–20 years, 13 at prepubertal, and 35 at pubertal and postpubertal ages, were consecutively recruited from outpatient Genetics Clinic at Mansoura University Children Hospital (MUCH) and Endocrinology Clinic at Specialized Medical Hospital, Mansoura University, Egypt (Table 1). The diagnosis of TS was based on standard karyotyping. All participants, patients, and parents signed an informed consent to be included in our study. The protocol study was approved by the local ethical committee.

### 2.1. Conventional Cytogenetic Analysis

Chromosomal cultures were set up according to G-banding [13]; about 1 mL of blood was mixed with 5 mL of RPMI medium, 1 mL of fetal bovine serum, and 0.1 *μ*g/mL of phytohemagglutinin (PHA) and incubated at 37°C. After 72 hours of incubation, the Colcemid (1 mg/mL) was added and incubated for another 1.5 hours. The cells were then harvested by hypotonic treatment (1.5 hours with 0.075 M KCl at 37°C), fixed, and washed thrice with fixative solution (methanol and acetic acid in a ratio of 3 : 1), and then metaphases were spread and stained using standard G-banding technique. For each case, 50 spread metaphases were analyzed with cytoVision system. 

### 2.2. Polymerase Chain Reaction

Genomic DNA was extracted from 2-3 mL ethylenediamine tetraacetate-containing blood using a standard Promega (Wizard Genomic DNA Purification Kit). Sets of oligonucleotide primers were used: SRY 1F 5′-CAG TGT GAA ACG GGAGAA AAC AGT-3′/SRY 2R 5′-CTT CCG ACG AGGTCG ATA CTT ATA-3′, which amplify a 270 bp fragment (518–788 bp) that mainly encompasses the HMG-box domain, an evolutionary highly conserved motif that codes for a protein with DNA-binding characteristics [14]. The primers were synthesized in (Eurofins, MWG/Operon) Amplification of a 165 bp fragment of the angiotensinogen gene was used as controls. The sequences of the primer pairs used to amplify the internal control were F 5′-CAG GGT GCT GTC CAC ACT GGACCC C-3′/R 5′-CCG TTT GTG CAG GGC CTGGCT CTC T-3′.

The PCR amplification was performed in a final volume of 50 *μ*L, the reaction mixture consisting of 300–500 ng genomic DNA, 50 pmol of each specific primer, and 30 pmol of the control primers and Master Mix Ready to be used with 7.5 mM MgCl_2_ (Solis Bio Dyne 5XHot FIRE Poll Blend). The amplification was carried out with a DNA thermal cycler (G-STORM GS482, By Gene Technologies, UK), 33 cycles for (SRY 1F/SRY 2R: 94°C, 10 min., 94°C, 45 seconds, 58°C, 1 min., and 72°C, 2 min.). Amplification conditions were based on a method previously reported [14] and self-adjusted temperatures.

All PCR products (10 *μ*L) were electrophoresed on a 2% agarose gel in 1XTBE buffer stained by ethidium bromide and visualized under UV light. Several precautions were taken to avoid false positive results [15]. All laboratory procedures were performed by a female operator, thus excluding the possibility for sample contamination with male cells. Pre- and post-PCR work spaces were strictly separated so that carryover of amplified DNA sequences to new PCR reactions was prevented. Each PCR reaction contained one normal female and one template-free sample for early detection of contamination. Each PCR reaction included one normal male sample as a positive control.

## 3. Results

### 3.1. Characteristics of Turner's Syndrome Patients

All TS patients were phenotypically females with short stature and primary amenorrhea. The other features encountered in TS patients were secondary sexual characteristics in 25.7% (9/35), dysmorphic features in 70.8% (34/48), cardiac anomalies and bicuspid aortic valve in 6.25% (3/48), hypoplastic uterus and nonvisualized ovaries in 56.25% (27/48), and rudimentary uterus with bilateral streak ovaries in 20.8% (10/48). None of TS patients had ambiguous genitals, neither features of virilization nor renal anomalies. Except for prepubertal patients, the basal levels of gonadotrophins suggested that severe gonadal failure (FSH > 60 mIU/mL) was present in 34.3% (12/35) (Table 1).

### 3.2. Cytogenetic and Molecular Findings in the Studied 48 Turner's Syndrome Patients

Conventional cytogenetic analysis identified classical 45,X karyotype in 50%, 45,X/46,XX in 18.75%, 45,X/46,X,i(Xq) in 12.5%, 46,XX/46,X,i(Xq) in 12.5%, and isochromosome 46,X,i(Xq) in 6.25%; none of TS patients had a cytogenetically obvious Y chromosome (Table 2). 

**Figure 1:**
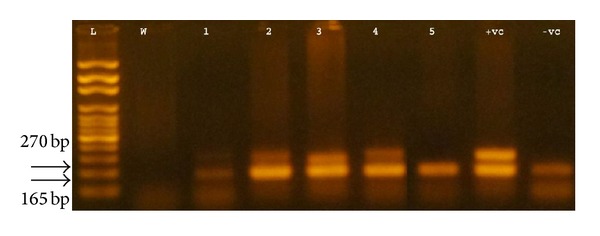
Agarose gel electrophoresis of the SRY gene PCR products from Turners syndrome patients amplified with SRY 1F/SRY 2R primers. Lane 1 (L): 100 bp DNA ladder 100–2000 bp (Amersham Pharmacia Biotech Inc., Piscataway, NJ, USA), lane 2 (W): template-free sample (negative control), lanes 3–7: patients 1–5 with various karyotypes, lane 8 (+vc): normal male sample (positive control), and lane 9 (−vc): normal female sample (negative control for SRY gene).

SRY gene was detected in 9 TS patients (18.75%) who had the classical 45,X karyotype, whereas all other patients were SRY negative (Table 2).  Figure 1 shows electrophoresis of PCR amplification products of a 270 bp SRY gene specific fragment using primers SRY 1F/SRY 2R.

## 4. Discussion

TS is characterized by a range of clinical stigmata, in which final height and gonadal function are almost always affected. Although conventional cytogenetic analysis is the definitive diagnosis of TS, it alone cannot be used to identify Y chromosome material or SRY gene; therefore molecular evaluation of Y-derivative sequences is useful to search for low frequency or hidden Y mosaicisms in 45,X karyotypes [12, 16]. PCR based analysis is fast, cheap, and easily feasible for screening TS patients.

There is controversy regarding the prevalence of hidden Y chromosome-derived material in TS patients; the present study was conducted to establish the prevalence of hidden Y chromosome mosaicism in TS Egyptian patients and compare our results with those in the literature. 

In the present study, cytogenetic analysis revealed various karyotype presentations. The classical 45,X nonmosaic karyotype was identified in 50% of TS patients, which corresponds to findings of other reports (40–60%) [17–19]. However, Hook and Warburton [20] found that only 20.7% of karyotypes of 87 alive born TS patients were 45,X.

Next in frequency are mosaicisms 45,X/46,XX, 45,X/46,X,i(Xq) and isochromosome 46,X,i(Xq). Their relative frequencies in the series are 13%, 8%, and 7%, respectively [21]. According to Mendes et al. [22] mosaicism is present in 25% of TS karyotypes. It has been speculated that most TS patients with putative nonmosaic 45,X karyotype have an undetected mosaicism, based on an analysis which revealed that less than 1% of 45,X conceptions survive pregnancy [20]. Several investigators have postulated that, in 45,X patients, fetal survival needs mosaicism in at least some organ or tissue [20, 23]. 

None of our patients had a cytogenetically obvious Y chromosome. This is in contrast with Álvarez-Nava et al. [24] who detected Y chromosome mosaicism in about 5% of patients with TS; however Y chromosome material may be missed by the conventional method if it is present in only a few cells.

We detected SRY gene in 9 TS patients (18.75%) with the nonmosaic 45,X karyotype, whereas all other patients were SRY gene negative. These results are consistent with previous reports of high prevalence of SRY gene in TS patients [9, 25, 26]. Kocova et al. [27] reported that the SRY gene is present in about 33.3% of TS patients, using the Southern blot technique after PCR. Others have shown a variable percentage of SRY gene positive TS patients [28, 29].

According to López et al. [18], the difficulty in comparing frequency of Y chromosome-specific sequences or Y chromosome itself in patients with TS is due mainly to variability in the number of patients analyzed, frequency of mosaicism with a normal and abnormal X chromosome, number of cases with marker chromosomes, molecular methodology applied in each study, and Y chromosome-specific sequences studied.

Of interest, our results showed that SRY gene positive TS patients had classical 45,X karyotype, with no cytogenetically obvious Y chromosome. These findings confirm the most earliest reports demonstrating that some TS patients with a karyotype that does not contain Y chromosome material indeed have low level mosaicism [18, 27, 30, 31], whereas others have not found any evidence of hidden Y chromosome material [5]. 

In accordance with Reena et al. [32], the results of our study have suggested that mosaic patients with cell lines containing two X chromosomes are less likely to be positive for SRY gene or Y chromosomal material.

In the present study, none of TS patients had features of virilization. These results are parallel to those of Bianco et al. [33], who reported that the presence of SRY gene was not associated with virilization, thus reinforcing the idea that absence of this characteristic does not rule out the possibility of the presence of hidden Y chromosome fragments. 

Patients with SRY gene require special attention since the presence of Y chromosome correlates with 10–30% risk of developing gonadoblastoma or dysgerminoma later in life [10, 11]. Gonadoblastoma is an in situ malignancy of the dysgenetic gonad with variable ages of appearance and considerable malignancy potential; dysgerminoma and other malignant germ cell tumors can arise within the gonadoblastoma [11, 34]. Detection of SRY gene in patients with or without cytogenetically detected sex chromosome mosaicism has important clinical and therapeutic implications [29, 35]. 

So, analysis of SRY gene should be offered to all TS patients because of (1) increased risk of gonadoblastoma [10], (2) possibility of “low level hidden” mosaicism for a Y chromosome positive cell line in the gonads, variable ages of expression [23], (3) high malignancy potential of gonadoblastoma, and (4) necessity of timely referral for gonadectomy. 

## 5. Conclusion

Cytogenetically undetected Y chromosome mosaicism is common in TS patients; these data reinforce the need for adequate diagnosis of Y chromosome material in those patients. Molecular screening for Y chromosome-derived DNA should be routinely carried out in all TS patients. 

## Figures and Tables

**Table 1:** Characteristics of Turner's syndrome patients.

Characteristics	48 Turner's syndrome patients *n* (%)
Short stature	100% (48/48)
Dysmorphic features	70.8% (34/48)
Secondary sexual characteristics	25.7% (9/35)
Primary amenorrhea	100% (7/7)
Ambiguous genitals	[−]
Male pattern	[−]
Features of virilization	[−]
Renal anomalies	[−]
congenital heart disease	6.25% (3/48)
FSH > 60 mIU/mL	34.3% (12/35)
*Sonographic data *	
(i) Hypoplastic uterus and nonvisualized ovaries	56.25% (27/48)
(ii) Rudimentary uterus with bilateral streak ovaries	20.8% (10/48)
(iii) Normal uterus and ovaries	22.9% (11/48)

Data are expressed as numbers or percentages; [−] indicates negativity.

**Table 2:** Cytogenetic and molecular findings in the studied 48 Turner's syndrome patients.

Conventional cytogenetic analysis	*n* (%)	SRY gene	*n* (%)
45,X	24 (50%)	[+]	9 (18.75%)
45,X/46,XX	9 (18.75%)	[−]	[−]
45,X/46,X,i(Xq)	6 (12.5%)	[−]	[−]
46,XX/46,X,i(Xq)	6 (12.5%)	[−]	[−]
46,X,i(Xq)	3 (6.25%)	[−]	[−]

Data are expressed as numbers or percentages, [−] indicates negativity for the SRY gene, and [+] indicates positivity for the SRY gene.
